# Supplementary material for: Exploring the Potential of Linear π-Bridge Structures in a D-π-A Organic Photosensitizer for Improved Open-Circuit Voltage
Source: Nanomaterials (Basel). 2024 Jun 27;14(13):1106. doi: 10.3390/nano14131106 (PMC11242973; doi:10.3390/nano14131106)
Supplement: Supplementary file 1 [file nanomaterials-14-01106-s001.zip › nanomaterials-3053094-supplementary.pdf]

Exploring the Potential of Linear π-Bridge Structures in D-π-A Organic Photosensitizer for Improved Open-Circuit Voltage

Min-Woo Lee [1,†], Seunghyun Yoo [2,†] and Chang Woo Kim [3,*]

1 Department of Chemistry, Sogang University, Seoul 04107, Republic of Korea; yohan-20@hanmail.net
2 R&D Team, The Day1Lab, #1007 Mario Tower, 28 Digital-ro 30-gil, Guro-gu, Seoul 08389, Republic of Korea; shyoo@day1-lab.com
3 Department of Nanotechnology Engineering, College of Engineering, Pukyong National University, Busan, 48513, Republic of Korea; kimcw@pknu.ac.kr
† These two authors contributed equally to this work
* Correspondence: kimcw@pknu.ac.kr

1. *Synthesis of organic photosensitizer, $_{sh}$PS, (Z)-3-(7-(Bis(9,9-dimethyl-9H-fluoren-2-yl)amino)-9,9-dimethyl-9H-fluoren-2-yl)-2-cyanoacrylic acid*

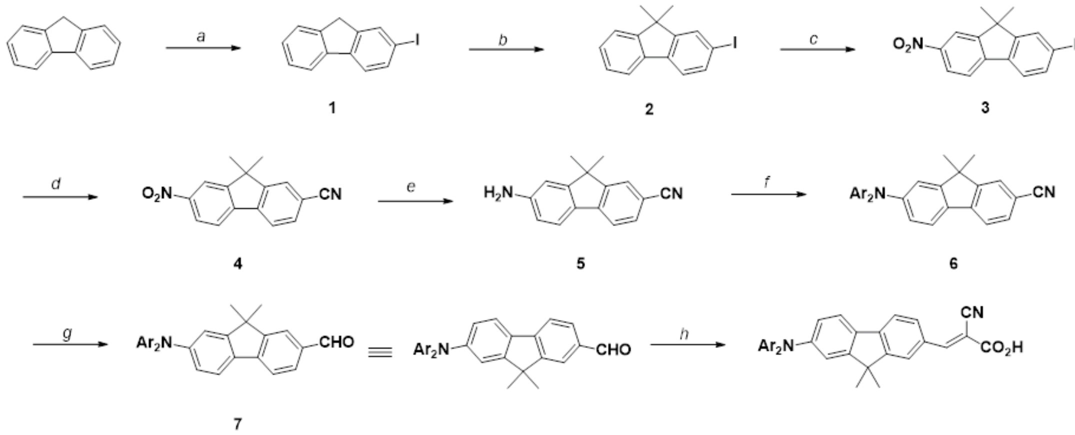

Scheme S1. Schematic illustration of synthetic process of $_{sh}$PS.

***Reagents and Conditions*:** (a) I_2 (0.34 eq), HIO_4 (0.17 eq), AcOH-H_2O-H_2SO_4 (100: 20: 3), reflux, 12 h, 51%. (b) KI (0.2 eq), CH_3I (10 eq), KOH (10 eq), DMSO, rt, 12 hr, 91%. (c) 90% HNO_3 (4 eq), HOAc (0.5 M), 80 °C, overnight, 81% (d) i) CuCN (1.2 eq), DMF (1.68 eq), reflux, overnight. ii) KCN (15 eq), H_2O, rt, 1h, 99% (e) $SnCl_2$-$2H_2O$ (5 eq), EtOH, 70 °C, 1 h, 88% (f) Ar-I(2) (2.5 eq), Cu (4 eq), K_2CO_3 (8 eq), 18-crown-6 (0.2 eq), dichlorobenzene, reflux, 2 d, 68% (g) DIBALH (0.3 M), DCM , -78 °C → -30°C, overnight, 78% (h) cyanoacetic acid (2 eq), piperidine (1 eq), CH_3CN, reflux, overnight, 48%.

(a) Fluorene (0.3 g, 1.80 mmol) was dissolved in the boiling solvent (CH_3COOH: H_2O: H_2SO_4 = 100: 20: 3, 6.05 mL) with stirring and the solution was cooled to 60-65 °C. After addition of periodic acid (70 mg, 0.31 mmol) and iodine (155 mg, 0.62 mmol), the reaction mixture was refluxed for 12 h and cooled to room temperature. The pale-yellow solid was collected by filtration, and washed with 2 M aqueous Na_2CO_3 and water. The crude product was purified by recrystallization from hexane to give **a white solid 1** (27 mg, 51 %). ^{1}H NMR ($CDCl_3$, 300 MHz) δ 7.89(s, 1H), 7.80~7.68(m, 2H), 7.53 (d, J = 6.90 Hz,

2H), 7.40~7.31(m, 2H), 3.87 (s, 2H); ^{13}C NMR ($CDCl_3$, 125 MHz) δ 207.2, 145.6, 142.8, 141.9, 140.9, 135.9, 134.3, 127.5, 127.1, 125.2, 121.7, 120.2, 36.8; GC/mass (m/z) calcd. for $C_{13}H_9I$ (M+) 291.97, found : 292.

(b) To a solution of 2-iodo-9H-fluorene, **a white solid 1** (1.3 g, 4.45 mmol) and potassium iodide (150 mg, 0.89 mmol) in DMSO (15 mL) were added iodomethane (6.39 g, 45.0 mmol) and potassium hydroxide (2.5 g, 45.0 mmol). The reaction mixture was stirred at room temperature for 12 h. The organic layer was separated and the aqueous layer extracted with ethyl acetate. The combined organic phases were washed with brine, dried with $MgSO_4$, and concentrated *in vacuo*. The crude residue was purified by column chromatography (hexane, $R_f = 0.5$) to give **adduct 2** (1.29 g, 91 %) as a yellow oil. ^1H NMR ($CDCl_3$, 300 MHz) δ 7.89(s, 1H), 7.80~7.68(m, 2H), 7.53 (d, J = 6.90 Hz, 2H), 7.40~7.31(m, 2H), 3.87 (s, 2H); ^{13}C NMR ($CDCl_3$, 125 MHz) δ 156.0, 153.1, 139.0, 138.3, 136.3, 136.1, 132.2, 128.0, 127.3, 122.7, 121.9, 120.2, 47.1, 27.1; GC/mass (m/z) calcd. for $C_{15}H_{13}I$ (M+) 320.01, found : 320.

(c) A mixture of 9,9-dimethyl-2-iodofluorene, **adduct 2** (1.0 g, 3.12 mmol), 6.2 mL of acetic acid, and 0.6 mL of 90 % nitric acid was heated slowly to reflux, which was maintained for 12 h. The mixture turned dark, then light, and was allowed to cool to 20 °C before being quenched in 10 mL of water with propeller stirring. The crude product was collected, washed with 50 mL of water and a minimum of 50 % methanol, dried at 60 °C/20 Torr/3 h to give (923 mg, 81 %) of **product 3**. IR (neat, cm^{-1}) 1509.83, 1339.97, 1136.75, 808.93, 733.42, 646.78; ^1H NMR ($CDCl_3$, 300 MHz) δ 8.27(s, 1 H), 8.24(d, J = 6.0 Hz, 1 H), 7.83(s, 1 H), 7.76(m, 2 H), 7.53(d, J = 6.1 Hz, 1 H), 1.53(s, 3 H); ^{13}C NMR ($CDCl_3$, 500 MHz) δ 157.15, 154.18, 147.71, 144.85, 136.89, 136.58, 132.71, 123.68, 123.12, 120.48, 118.49, 95.66, 47.64, 26.79; LC/MS (m/z) calcd. for $C_{15}H_{12}INO_2$ (M+) 364.99, found : 366.19.

(d) A suspension of 9,9-dimethyl-2-iodo-7-nitrofluorene, **product 3** (500 mg, 1.19 mmol), copper(I) cyanide (128 mg, 1.42 mmol), and 2.0 mL DMF was boiled under reflux overnight and allowed to cool to about 50 °C, then poured into a solution of potassium cyanide (294 mg, 18.6 mmol) in 20 mL of water with propeller stirring. During the course of an hour the gummy solid turned hard and was collected, washed with 10 mL of water, ground in a mortar, slurred with 50 mL of 47.5 % ethanol, re-filtered, washed on the filter with 100 mL of 47.5 % ethanol, and then dried at 80 °C/25 Torr/2 h to give 42.3 g. This was extracted from a glass thimble in a large Soxhlet with 100 mL of cyclohexane and the extract was transferred to a beaker, diluted with 50 mL of hexane, and allowed to stand at 20 °C overnight to yield nice tan **spars 4** (312 mg, 99%). IR (neat, cm^{-1}) 2222.87, 1516.30, 1341.62, 889.36, 823.37, 789.81, 735.99; ^1H NMR ($CDCl_3$, 300 MHz) δ 8.33(d, 1 H), 8.30(d, 1 H), 7.89(d, 2 H), 7.77(d, 1 H), 7.72(m, 1 H), 1.57(s, 3 H); ^{13}C NMR ($CDCl_3$, 500 MHz) δ 155.54, 155.33, 143.52, 141.30, 131.94, 126.97, 123.76, 122.17, 121.60, 119.09, 118.66, 112.47, 47.91, 26.63; GC/MS (m/z) calcd. for $C_{16}H_{12}N_2O_2$ (M+) 264.09, found : 264.

(e) A mixture 4 (400 mg 1.51mmol) and (1.7 g 7.57 mmol) of $SnC1_2$-$2H_2O$ in 20 mL of absolute ethanol is heated at 70 °C under nitrogen. After 1 h the starting material has disappeared and the solution is allowed to cool down and then poured into ice. The pH is made slightly basic (pH 7-8) by addition of 5 % aqueous sodium bicarbonate (8) before being extracted with ethyl acetate. The organic phase is thoroughly washed with brine, treated with charcoal and dried over sodium sulfate.

Evaporation of the solvent leaves **5 (310 mg, 88 %) of solid product**. IR (neat, cm^{-1}) 2221.97, 1626.21, 1602.72, 1467.49, 1444.20, 1351.47, 1314.96, 1293.80, 1169.92, 824.97, 739.33, 643.76, 607.90; ^{1}H NMR ($CDCl_3$, 300 MHz) δ 7.52~7.61(m, 4 H), 6.74(d, J = 6.0 Hz, 1 H), 6.68(m, 1 H), 3.91(s, 2 H), 1.44(s, 3 H); ^{13}C NMR ($CDCl_3$, 500 MHz) δ 156.46, 153.22, 148.11, 144.55, 131.57, 127.91, 126.01, 122.29, 120.29, 118.98, 114.38, 109.00, 107.77, 46.81, 26.97; LC/MS (m/z) calcd. for $C_{16}H_{14}N_2$ (M+) 234.12, found : 235.10.

(f) A mixture of 2-iodo-9,9-dimethyl-9H-fluorene 2 (3.4 g, 10.67 mmol), potassium carbonate (4.7 g, 34.14 mmol), activated powder copper (1.08 g, 17.07 mmol), 18-crown-6 (0.23 g, 0.85 mmol), **solid product 5** (1.0 g, 4.27 mmol) and 1,2-dichlorobenzene (8.5 mL) was kept in reflux for 12 h under N_2 flow, cooled and filtered. After concentration of the filtrate, the residue solid was purified by column chromatography (silica gel, ethyl acetate : hexane = 1 : 5, R_f = 0.48) to give **product 6** as a white solid (1.8 g, 68 %). IR (neat, cm^{-1}) 2221.84, 1603.27, 1461.33, 1347.68, 818.28, 757.47, 736.10; ^{1}H NMR ($CDCl_3$, 300 MHz) δ 763~7.72(m, 9 H), 7.30~7.45(m, 7 H), 7.16~7.21(m, 4 H), 1.46(d, J = 6.0 Hz, 18 H); ^{13}C NMR ($CDCl_3$, 500 MHz) δ 155.86, 155.29, 154.03, 153.66, 149.51, 147.16, 143.97, 138.98, 134.84, 131.75, 131.43, 127.23, 126.84, 126.34, 123.73, 122.70, 122.59, 121.94, 120.88, 120.13, 119.79, 119.66, 119.20, 117.41, 108.91, 47.22, 47.02, 27.19, 26.85; LC/MS (m/z) calcd. for $C_{46}H_{38}N_2$ (M+) 618.2, found : 618.2.

(g) In a 50 mL three-necked flask fitted with a septum inlet, a low-temperature thermometer, and a septum outlet **product 6** (1.0 g, 1.62 mmol) was dissolved in dry dichloromethane (10 mL). The solution was cooled to -78 °C, where upon DIBAL-H (4.85 mL, 1 M solution in hexanes) was added dropwise by means of a syringe. The solution was allowed to warm to -30 ℃ over a period of 12 h. Hydrolysis was effected slowly with a homogeneous mixture of 8.6 g silica gel and 2.6 mL water. After stirring for 1 h at 0 ℃, K_2CO_3 and $MgSO_4$ were added, the solids were filtered off and rinsed thoroughly with dichloromethane and diethyl ether. The solvents were evaporated and column chromatography (ethyl acetate : hexane = 1 : 5, R_f = 0.4) to give product as **a white solid 7** (787 mg, 78 %). IR (neat, cm^{-1}) 1695.64, 1600.83, 1459.20, 1449.26, 1348.25, 1320.35, 1300.14, 819.45, 756.05, 734.78, 648.18; ^{1}H NMR ($CDCl_3$, 300 MHz) δ 10.02(s, 1 H), 7.91(s, 1 H), 7.84(d, J = 6.0 Hz, 1 H), 7.75(d, J = 6.2 Hz, 1 H), 7.65(m, 5 H), 7.40(d, J = 6.4 Hz, 2 H), 7.34(d, J = 6.1 Hz, 2 H), 7.28(m, 5 H), 7.14(m, 3 H), 1.43(d, J = 6.8 Hz, 18H); ^{13}C NMR ($CDCl_3$, 500 MHz) δ 192.25, 156.68, 155.28, 154.22, 153.68, 149.44, 147.22, 145.86, 139.01, 134.80, 134.77, 131.87, 131.10, 127.23, 126.82, 123.73, 122.97, 122.71, 122.62, 122.11, 120.88, 119.66, 119.54, 119.20, 117.56, 47.03, 27.20, 26.96; LC/MS (m/z) calcd. for $C_{46}H_{39}NO$ (M+) 621.3, found : 621.63.

(h) To a mixture of aldehyde, **a white solid 7** (450 mg, 0.724 mmol) and cyanoacetic acid (123 mg, 1.45 mmol) were added acetonitrile (3.6 mL) and piperidine (61.6 mL, 0.724 mmol) at room temperature. The solution was refluxed overnight. After cooling to room temperature, the organic phase was separated and the aqueous layer extracted with CH_2Cl_2. The combined organic phases were washed with brine, dried with $MgSO_4$, and concentrated *in vacuo*. The crude residue was purified by column chromatography (methanol:dichloromethane = 1 : 10, R_f = 0.17) to give **$_{sh}$PS** (240 mg, 48 %) as a red solid.

IR (neat, cm^{-1}) 1600.68, 1462.35, 1311.90, 758.77, 736.23, 651.67, 628.97; ^{1}H NMR (DMSO-d_6, 300 MHz) δ 8.00 (d, J = 6.0 Hz, 1 H), 7.83(m, 2 H), 7.76(m, 5 H), 7.51(d, J = 6.6 Hz, 2 H), 7.29(m, 8 H), 7.04(m, 3 H), 1.37(s, 18 H); ^{13}C NMR (DMSO-

d6, 500 MHz) δ 164.03, 155.61, 154.75, 153.47, 153.14, 149.03, 148.03, 146.65, 141.51, 138.24, 134.00, 132.11, 131.08, 129.18, 127.08, 126.70, 123.98, 123.13, 122.67, 122.16, 122.01, 121.16, 119.85, 119.56, 119.25, 118.53, 117.22, 110.19, 62.81, 46.42, 26.64, 26.48; LC/MS (m/z) calcd. for $C_{49}H_{42}N_2O_2$ (M+) 688.31, found : 688.91.

2. *Synthesis of 6-bromobenzo[b]thiophene-2-carbaldehyde*

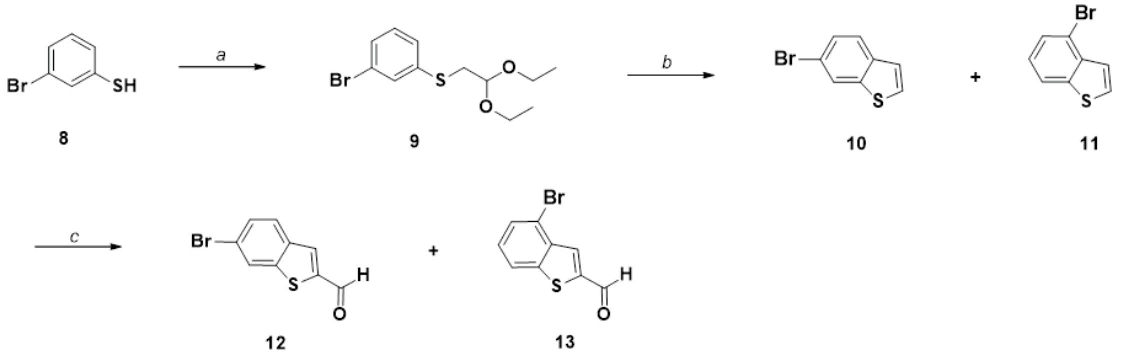

Scheme S2. Schematic illustration of synthetic process of **bromoaldehyde 12** and **13**.

***Reagents and Conditions*:** (a) 2-bromo-1,1-diethoxyethane (55 eq), NaH (59 eq), THF, rt to reflux, 100% (b) polyphosphoric acid (2 eq), chlorobenzene, reflux, 36%. (c) i) diisopropylamine (14.17 mmol), ii) n-BuLi (9.77 mmol, 1.6 eq), iii) DMF (2.3 eq), THF, 60%.

(a) 3-Bromobenzothiol 8 (5 mL, 48.13 mmol) was dissolved in 250 mL fresh distilled THF; sodium hydride (1.42 g, 59.23 mmol, in 60 % mineral oil) was added in the flask. The mixture was stirred for 10 min at room temperature and then bromoacetaldehyde-dimethylacetal (8.47 mL, 55.0 mmol) was injected into the flask. Further, the reaction mixture was refluxed for 24 h. After solvent evaporation, the residue was dissolved in ethyl acetate (50 mL), washed with a saturated solution of $NaHCO_3$ (3×30 mL), and dried over Na_2SO_4. After filtration and evaporation of the solvent, the crude product was purified by column chromatography with 10 g of silica gel eluting with hexanes ⁄ dichloromethane (5:1) to give a pale green oil, **compound 9** (14.54 g, 99 %). IR (neat, cm^{-1}) 1122.8, 1058.0, 1015.9, 771.3, 678.09; ^{1}H NMR ($CDCl_3$, 300 MHz) δ 7.51(s, 1 H), 7.29(d, *J* = 6.4 Hz, 2 H), 7.10(t, J = 4.6 Hz, 1 H), 4.64(t, 1 H), 3.67(m, 2 H), 3.54(m, 2 H), 3.12(d, *J* = 6.2 Hz, 2 H), 1.19(t, J = 5.6 Hz, 6H); ^{13}C NMR ($CDCl_3$, 125 MHz) δ 139.37, 131.56, 130.34, 129.15, 127.63, 122.97, 102.01, 62.71, 62.57, 37.42, 15.50; GC/MS (m/z) calcd. for C_8H_5BrS (M+) 304.01, found : 304.

(b) Polyphosphoric acid (12.4 g) and cholorobenzene (150 mL) mixture was refluxed for 3 h and then **compound 9** in 50 mL of cholorobenzene (6.20 g, 20.3 mmol) was injected into the flask. The reaction mixture was refluxed for an additional 24 h while silicon oil temperature was maintained at 180 °C. Cholorobenzene was then evaporated *in vacuo* and the resulting residue dissolved in CH_2Cl_2 (50 mL). The solution was washed with $NaHCO_3$ solution (3×30 mL) and dried over Na_2SO_4. After

filtration and evaporation of the solvent, the crude product was purified by column chromatography with 10 g of silica gel eluting with hexanes to give **a pale yellow oil 10** and **11** (5.78 g, 65 %). IR (neat, cm^{-1}) 867.3, 816.4, 747.8, 690.4; ^{1}H NMR ($CDCl_3$, 300 MHz) δ d 7.96 (m, 1H), 7.74 (d, J = 8.4 Hz, 1H), 7.59 (d, J = 8.4 Hz, 1H), 7.49 (m, J1 = 0.8 Hz, J2 = 7.8 Hz, 1H), 7.44-7.40 (m, 3H), 7.34 (d, J = 5.6 Hz, 1H), 7.22 (m, J1 = 0.4,J2 = 5.6 Hz, 1H), 7.12 (t, J = 5.6 Hz, 1H); ^{13}C NMR ($CDCl_3$, 300 MHz): δ 141.3, 140.5, 139.5, 138.3, 127.6, 127.5, 127.3, 127.0, 125.2, 125.0, 124.7, 124.3, 123.6, 121.6, 118.2, 117.5; GC/MS (m/z) calcd. for C_8H_5BrS (M+) 211.93, found : 212.

(c) n-BuLi (3.94 mL, 2.48 M solution in hexane) was added to diisopropylamine (2 mL) in dry THF (15 mL) at 0 °C under N_2 and stirring at -78°Cfor 30 min, which then added dropwise to the mixture of **compound 10** or **11** (1.30 g, 6.10mmol) in dry THF (5 mL). Lastly DMF (1.09 mL, 14.3 mmol) was added to reaction mixture dropwise at -78 °C for 2 h. The reaction mixture was quenched aq.NH_4Cl and the organic layer was separated and the aqueous layer extracted with ethyl acetate. The combined organic phases were washed with brine, dried with $MgSO_4$, and concenetrated *in vacuo*. The crude residue was purified by column chromatography to give **product 12** and **13** (0.97 g, 66 %). IR (neat, cm^{-1}) 1674.6, 1585.2, 1132.9, 853.54, 666.4; ^{1}H NMR ($CDCl_3$, 300 MHz) δ 10.10(s, 1 H), 8.06(s, 1 H), 8.00(s, 1 H), 7.81(d, J = 6.4 Hz, 1 H), 7.57(d, 1 H); ^{13}C NMR ($CDCl_3$, 125 MHz) δ 184.6, 144.01, 143.93, 137.42, 133.91, 129.20, 127.42, 126.06, 122.85; GC/MS (m/z) calcd. for C_9H_5BrOS (M+) 239.92, found : 242.

3. *Synthesis of N-(9,9-Dimethyl-9H-fluoren-2-yl)-9,9-dimethyl-N-(4-(4,4,5,5-tetramethyl-1,3,2-di oxaborolan-2-yl) phenyl)-9H-fluore n-2-amine naphthalene*

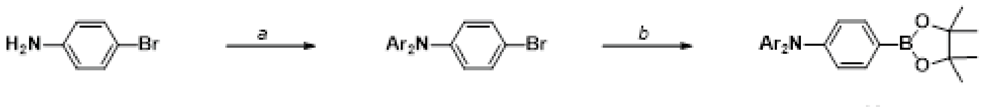

Scheme S3. Schematic illustration of synthetic process of **dioxaborolane 16**.

***Reagents and Conditions*:** (a) Ar-I(2) (2.5 eq), Cu (4 eq), K_2CO_3 (8 eq), 18-crown-6 (0.2 eq), dichlorobenzene, reflux, 12 h, 42% (b) i) n-BuLi (2.48M, 1.2eq), THF, 20min, ii) 2-isopropoxy-4,4,5,5-tetramethyl-1,3,2-dioxaborolane (1.3 eq), 73%.

(a) A mixture of 2-iodo-9,9-dimethyl-9H-fluorene **2** (11.3 g, 35.3 mmol), potassium carbonate (19.5 g, 35.3 mmol), activated powder copper (2.41 g, 37.9 mmol), 18-crown-6 (0.93 g, 3.53 mmol), 4-bromoaniline (3 g, 15.7 mmol) and 1,2-dichlorobenzene (88.2 mL) was kept in reflux for 12 h under N_2 flow, cooled and filtered. After concentration of the filtrate, the residue solid was purified by column chromatography (silica gel, hexane : toluene = 10 : 1, R_f = 0.4) to give product as **a white solid 15** (4 g, 40 %). IR (neat) : 1608, 1485, 1447, 820.7, 780.0; ^{1}H NMR($CDCl_3$, 300 MHz): δ 7.66 - 6.96 (m, 18 H), 1.40 (s, 12 H); ^{13}C NMR ($CDCl_3$, 125 MHz): δ 139.01, 138.25, 134.73, 134.63, 132.36, 129.22, 128.41, 127.20, 126.78, 125.70,

125.50, 123.44, 123.31, 122.68, 120.86, 119.66, 118.80, 47.02, 27.21; LC/MS (m/z) calcd. $C_{36}H_{30}BrN$ (M+) 555.16, found: 555.44.

(b) n-BuLi (2.48 M, 2.17 mL) was added dropwise to a solution of give *N*-(4-bromophenyl)-*N*-(9,9-dimethyl-9H-fluoren-2-yl)-9,9-dimethyl -9H-fluoren-2-amine **15** (2 g, 3.59 mmol) in THF (36 mL) at -78 °C and 2-isopropoxy-4,4,5,5-tetramethyl-1,3,2-dioxaborolane (0.95 mL, 4.67 mmol) was added at -78 °C. The reaction mixture was stirred at -78 °C for 20 min. The organic layer was separated and the aqueous layer extracted with ethyl acetate. The combined organic phases were washed with brine, dried with Na_2SO_4, and concentrated *in vacuo*. The crude residue was purified by column chromatography (ethyl acetate : Hexane = 1:20, $R_f = 0.5$) to give adduct **16** (1.6 g, 73%) off-white solid.

IR (neat, cm^{-1}) 1599, 1361, 1145, 1093, 830; ^1H NMR ($CDCl_3$, 400 MHz) δ 7.67(d, J=8Hz, 2H), 7.63(d, J=4Hz, 2H), 7.57(d, J=8Hz, 2H), 7.37(d, J=4Hz, 2H), 7.31-7.26(m, 3H),7.22(d, J=4Hz, 2H), 7.16(d, J=4Hz, 1H), 7.14(s, 1H), 7.12(s, 1H), 7.07(d, J=4Hz, 2H), 1.38(s, 12H), 1.33(s, 12H); ^{13}C NMR ($CDCl_3$, 400 MHz) δ 155.1, 153.7, 150.8, 147.0, 139.0, 136.0, 134.7, 127.1, 126.7, 123.8, 122.6, 122.1, 120.7, 119.6, 119.3, 83.76, 46.98, 27.16, 25.0; LC/MS: (m/z) calcd. for $C_{42}H_{42}BNO_2$ (M+) 603.33 found: 603.37.

4. *Synthesis of organic dye, cPS, (Z)-3-(4-(4-(bis(9,9-dimethyl-9H-fluoren-2-yl)amino) phenyl) benzo [b]thiophen-2-yl)-2-cyanoacrylic acid*

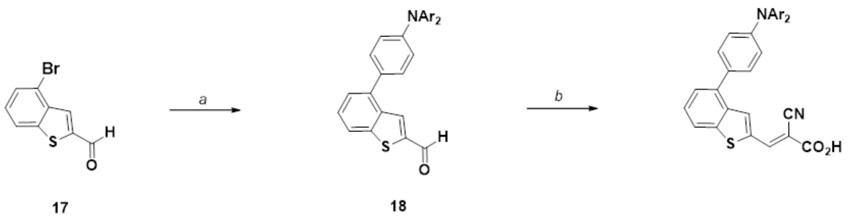

Scheme S4. Schematic illustration of synthetic process of $_c$PS.

***Reagents and Conditions*:** (a) **16** (1.1 eq), $Pd(PPh_3)_2Cl_2$ (0.09 eq), 2 M Na_2CO_3-DME-H_2O (5 : 9 : 1), 100 ºC, overnight, 56%. (b) $NCCH_2CO_2H$ (2 eq), piperidine (1 eq), CH_3CN, reflux, overnight, 95%.

(a) A mixture of 4-bromobenzo[b]thiophene-2-carbaldehyde **17** (160 mg, 0.66 mmol), N-(9,9-dimethyl-9H-fluoren-2-yl)-9,9-dimethyl-N-(4-(4,4,5,5-tetramethyl-1,3,2-dioxaborolan-2-yl)phenyl)-9H-fluoren-2-amine **16** (508mg, 0.86 mmol) and $Pd(PPh_3)_2Cl_2$ (42 mg, 0.06 mmol) in 2 M aqueous Na_2CO_3 solution (1.2 mL), H_2O (0.25 mL) and dimethoxyethane (2.21 mL) was stirred at 100 °C overnight. After cooling to room temperature, the reaction mixture was filtered through celite, and the filtrate was poured into water and extracted with CH_2Cl_2. The combined organic phases were washed with brine, dried with Na_2SO_4, and concentrated *in vacuo*. The crude residue was purified by column chromatography (ethyl acetate : hexane = 1 :

10, R_f = 0.5) to give **adduct 18** (250 mg, 56 %). ^{1}H NMR(DMSO-d_6, 300 MHz) δ 10.16(s, 1 H), 8.51(s, 1 H), 8.01(s, 1 H), 8.10(d, J = 6.1Hz, 1 H), 7.82~7.58(m, 4 H), 7.68~7.59(m, 5H), 7.54~7.51(m, 4H), 7.35~7.22(m, 7H), 7.14~7.12(m, 2H), 1.39(s, 12H); ^{13}C NMR ($CDCl_3$, 125 MHz) δ 184.70, 115.33, 153.71, 148.30, 147.17, 143.88, 143.34, 141.17, 139.05, 137.42, 134.73, 134.53, 133.67, 128.26, 127.21, 126.79, 126.65, 124.83, 123.80, 123.66, 122.71, 120.87, 120.81, 119.66, 119.14, 47.05, 27.23; LC/MS (m/z) calcd. $C_{45}H_{35}NOS$ (M+) 637.24, found : 636.81.

(b) To a mixture of aldehyde **18** (240 mg, 0.36 mmol) and cyanoacetic acid (61 mg, 0.72 mmol) were added acetonitrile (12.0 mL) and piperidine (0.035 mL, 0.72 mmol) at room temperature. The solution was refluxed overnight. After cooling to room temperature, the organic phase was separated and the aqueous layer extracted with CH_2Cl_2. The combined organic phases were washed with brine, dried with $MgSO_4$, and concentrated in vacuo. The crude residue was purified by column chromatography (ethyl acetate : methanol = 1 : 1, R_f = 0.8) to give adduct $_c$**PS** (242 mg, 95 %) as a red solid. ^{1}H NMR(DMSO-d_6, 300 MHz) δ 8.72(s, 1 H), 8.50(s, 1 H), 8.11(d, J = 6Hz, 1 H), 7.80(d, J = 6Hz, 2H), 7.76(d, J = 6Hz, 2H), 7.63~7.57(m, 4H), 7.51(d, J = 6Hz, 3H), 7.33~7.25(m, 7H), 7.21(d, J = 6Hz, 2H), 7.13(d, J = 6Hz, 2H), 1.38(s, 12H); ^{13}C NMR (DMSO-d_6, 125 MHz) δ 163.32, 154.83, 153.17, 147.33, 146.51, 142.53, 138.73, 138.23, 136.80, 136.41, 134.26, 134.06, 132.94, 127.99, 127.08, 126.72, 125.64, 124.03, 123.22, 122.70, 121.21, 121.15, 119.63, 119.56, 118.59, 117.55, 46.45, 43.05, 26.67, 22.20, 21.75; LC/MS (m/z) calcd. $C_{48}H_{36}N_2O_2S$(M+) 704.25, found : 703.91.

5. *Synthesis of organic dye, $_{lo}$PS, (E)-3-(6-(4-(bis(9,9-dimethyl-9H-fluoren-2-yl)amino)phenyl)benzo [b]thiophen-2-yl)-2-cyanoacrylic acid*

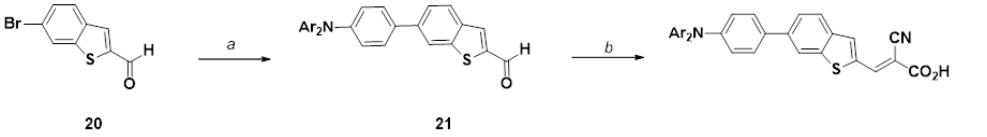

Scheme S5. Schematic illustration of synthetic process of $_{lo}$PS.

***Reagents and Conditions*:** (a) **16** (1.1 eq), $Pd(PPh_3)_2Cl_2$ (0.09 eq), 2 M Na_2CO_3-DME-H_2O (5 : 9 : 1), 100 ºC, overnight, 65%. (b) $NCCH_2CO_2H$ (2 eq), piperidine (1 eq), CH_3CN, reflux, overnight, 33%.

(a) A mixture of 6-bromobenzo[b]thiophene-2-carbaldehyde **20** (120 mg, 0.50 mmol), N-(9,9-dimethyl-9H-fluoren-2-yl)-9,9-dimethyl-N-(4-(4,4,5,5-tetramethyl-1,3,2-dioxaborolan-2-yl)phenyl)-9 H-fluoren-2-amine **16** (450 mg, 0.75 mmol) and $Pd(PPh_3)_2Cl_2$ (31.39 mg, 0.045 mmol) in 2 M aqueous Na_2CO_3 solution (1.38 mL), H_2O (0.28 mL) and 1,2-dimethoxyethane (2.48 mL) was stirred at 100 °Covernight. After cooling to room temperature, the reaction mixture was filtered through celite, and the filtrate was poured into water and extracted with CH_2Cl_2. The combined organic phases were washed with brine, dried

over Na_2SO_4, and concentrated in vacuo. The crude residue was purified by column chromatography (ethyl acetate : hexane = 1 : 8, R_f = 0.5) to give adduct **21** (206 mg, 65 %). IR (neat, cm^{-1}) 1672.2, 1597.3,1503.4, 1448.0, 1314.2, 1133.7, 736.5; ^1H NMR($CDCl_3$, 300 MHz) δ 10.11(s, 1 H), 8.12(s, 1 H), 8.01(s, 1 H), 7.98(d, J = 6.2Hz 1 H), 7.73~7.13(m, 18 H), 1.43(s, 12 H); ^{13}C NMR ($CDCl_3$, 125 MHz) δ 184.70, 115.33, 153.71, 148.30, 147.17, 143.88, 143.34, 141.17, 139.05, 137.42, 134.73, 134.53, 133.67, 128.26, 127.21, 126.79, 126.65, 124.83, 123.80, 123.66, 122.71, 120.87, 120.81, 119.66, 119.14, 47.05, 27.23; LC/MS (m/z) calcd. $C_{45}H_{35}NOS$ (M+) 637.24, found : 636.81.

(b) To a mixture of aldehyde **21** (144 mg, 0.23 mmol) and cyanoacetic acid (38.3 mg, 0.45 mmol) were added acetonitrile (2.30 mL) and piperidine (19.2 mg, 0.23 mmol) at room temperature. The solution was refluxed overnight. After cooling to room temperature, the organic phase was separated and the aqueous layer extracted with CH_2Cl_2. The combined organic phases were washed with brine, dried with $MgSO_4$, and concentrated in vacuo. The crude residue was purified by column chromatography (ethyl acetate : methanol = 1 : 1, R_f= 0.7) to give adduct $_{\mathbf{lo}}$**PS** (80 mg, 51 %) as a red solid

^1H NMR(DMSO-d_6, 300 MHz) δ 8.67(s, 1 H), 8.49(s, 1 H), 8.35(s, 1 H), 8.10(d, J = 6.8Hz, 1 H), 7.86~7.75(m, 19 H), 1.38(s, 12 H); ^{13}C NMR (DMSO-d_6, 125 MHz) δ 163.32, 154.83, 153.17, 147.33, 146.51, 142.53, 138.73, 138.23, 136.80, 136.41, 134.26, 134.06, 132.94, 127.99, 127.08, 126.72, 125.64, 124.03, 123.22, 122.70, 121.21, 121.15, 119.63, 119.56, 118.59, 117.55, 46.45, 43.05, 26.67, 22.20, 21.75; LC/MS (m/z) calcd. $C_{48}H_{36}N_2O_2S$ (M+) 704.25, found : 703.91.

6. *Synthesis of organic dye, $_t$PS, (E)-3-(5-(4-(bis(9,9-dimethyl-9H-fluoren-2-yl)amino)phenyl) naph tha[1,2-b:4,3-b′]dithiophen-2-yl)-2-cyanoacrylic acid*

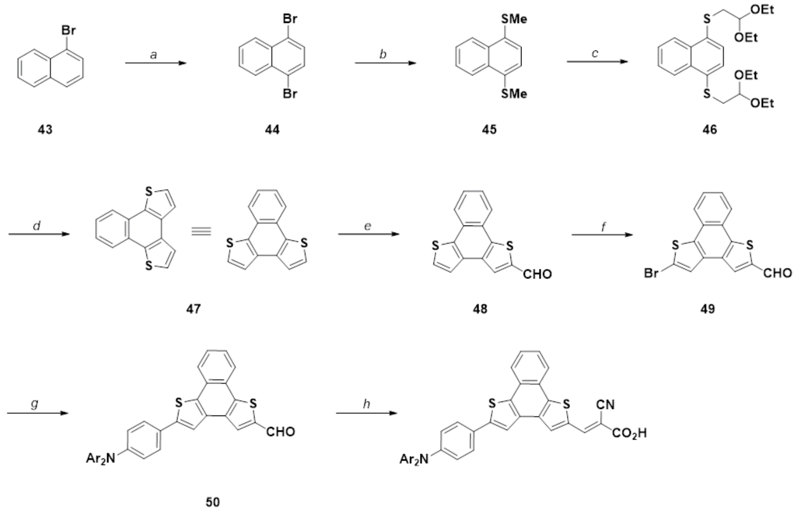

Scheme S6. Schematic illustration of synthetic process of $_t$**PS**.

***Reagents and Conditions*:** (a) Br_2 (1.1 eq), DCM, -30 °C, 2 d, 85% (b) MeSSMe (6 eq), n-BuLi (2.31 M, 0.4 eq), THF, rt, 12 h, 53% (c) i). Na (7 eq), DMA, 100 °C, 24 h. ii) Bromoactaldehydediethylacetal (4 eq), 100 °C, 24 h, 60%, (d) Polyphosphoric acid (2 eq), cholorobenzene, reflux, 24 h, 88% (e) n-BuLi (2.25 M, 1.2 eq), TMEDA (2 eq), DMF (1.5 eq), THF, -78°C, 12 h, 80% (f) NBS (3 eq), MC, rt, 3 h, 77%. (g) **16** (1.1 eq), $Pd(PPh_3)_2Cl_2$ (0.09 eq), 2 M Na_2CO_3-DME-H_2O (5 : 9 : 1), 100 ºC, overnight, 67%. (h) $NCCH_2CO_2H$ (2 eq), piperidine (1 eq), CH_3CN, reflux, overnight, 59%.

(a) The solution of 1-bromonaphthalene **43** (10 g, 48.29 mmol) in CH_2Cl_2 (48 mL) was cooled at -30 °C. To the solution was added initially cooled (-30 °C) Br_2 (2.7 mL, 53.12 mmol) in CH_2Cl_2 (10 mL). The reaction mixture was allowed to stand in a freezer at -30 °C for 2 days in the dark. It was seen that some of the product recrystallized. After removal of the solvent at reduced pressure reaction material was dissolved in CH_2Cl_2 and diluted with petroleum ether (CH_2Cl_2: petroleum ether = 1:4) and allowed to stand in the freezer (-15 °C). Fractional crystallization in a few days gave pure product in a yield of **44** (11.7 g, 85 %). IR (neat, cm^{-1}) 1583.65, 1492.73, 1366.40, 1252.85, 1183.49, 960.83, 814.00, 751.84; ^1H NMR ($CDCl_3$, 300 MHz) δ 8.26(m, 2 H), 7.66(m, 2 H), 7.63(s, 2 H); ^{13}C NMR ($CDCl_3$, 500 MHz) δ 132.98, 130.13, 128.24, 127.84; GC/MS (m/z) calcd. for $C_{10}H_6Br_2$ (M+) 285.88, found : 286.

(b) A solution of n-BuLi (45.4 mL, 2.31 M solution in hexane) was added slowly to a solution of 1,4-dibromonaphthalene **44** (5 g, 17.48 mmol) in THF (25 mL) at -78 °C. After stirring for 1 h $(CH_3S)_2$ (9.44 mL, 104.91 mmol) was added to the reaction mixture and stirred for another 1 h. The resulting mixture was extracted with ether (3 x 20 mL). The combined organic phases were dried over Na_2SO_4. The volatile components were removed *in vacuo* to afford a pale yellow residue that was purified by column chromatography, eluting with ethyl acetate : hexane = 1:10. Recrystallization from ethyl acetate : hexane = 1:10 produced white crystals of **45** (2.04 g, 53 %). IR (neat, cm^{-1}) 1434.39, 1365.62, 1200.34, 992.21, 812.77, 752.44; ^1H NMR ($CDCl_3$, 300 MHz) δ 8.33(m, 2 H), 7.58(m, 2 H), 7.38(s, 2 H), 2.55(s, 6 H); ^{13}C NMR ($CDCl_3$, 500 MHz) δ 133.84, 132.14, 126.68, 125.22, 124.55, 16.83; GC/MS (m/z) calcd. for $C_{12}H_{12}S_2$ (M+) 220.04, found : 220.

(c) 1,4-Bis(methylthio)naphthalene **45** (10 g, 45.4 mmol) was dissolved in dimethylacetamide (91 mL); sodium (7.3 g, 317.7 mmol) was added to the flask. The mixture was stirred for 24 h at 100 °C and then bromoacetaldehyde-dimethylacetal (28 mL, 181.5 mmol) was injected into the flask. Further, the reaction mixture was refluxed for 24 h. After solvent evaporation, the residue was dissolved in ethyl acetate (50 mL), washed with a saturated solution of $NaHCO_3$ (3×30 mL), and dried over Na_2SO_4. After filtration and evaporation of the solvent, the crude product was purified by column chromatography with 10 g of silica gel eluting with ethyl acetate : hexane = 1:5 to give a pale yellow oil, compound **46** (11.6 g, 60 %). IR (neat, cm^{-1}) 1370.63, 1121.34, 1057.55, 1014.95, 992.23, 759.68; ^1H NMR ($CDCl_3$, 300 MHz) δ 8.46(m, 2 H), 7.59(m, 2 H), 7.56(s, 2 H), 4.65(t, J = 8.8Hz, 2 H), 3.65(m, 4 H), 3.49(m, 4 H), 3.15(d, J = 6.8Hz, 4 H), 1.18(t, J = 6.2Hz, 12 H); ^{13}C NMR ($CDCl_3$, 300 MHz) δ 133.43, 133.08, 128.34, 126.81, 125.90, 101.61, 62.09, 38.02, 15.32; GC/MS (m/z) calcd. for $C_{22}H_{32}O_4S_2$ (M+) 424.17, found : 424.

(d) Polyphosphoric acid (2 g) and cholorobenzene (33 mL) mixture was refluxed for 3 h and then compound **46** in 7 mL of cholorobenzene (1 g, 2.36 mmol) was injected into the flask. The reaction mixture was refluxed for an additional 24 h while silicon oil temperature was maintained at 140 °C. Cholorobenzene was then evaporated in vacuo and the resulting residue dissolved in CH_2Cl_2 (50 mL). The solution was washed with $NaHCO_3$ solution (3 × 30 mL) and dried over Na_2SO_4. After filtration and evaporation of the solvent, the crude product was purified by column chromatography with 10 g of silica gel eluting with hexanes to give a pale yellow solid **47** (500 mg, 88 %). IR (neat, cm^{-1}) 1455.32, 1258.55, 844.21, 749.80, 716.09, 622.39; ^1H NMR ($CDCl_3$, 300 MHz) δ 8.19(m, 2 H), 7.66(d, 2 H), 7.58(m, 2 H); ^{13}C NMR ($CDCl_3$, 500 MHz) δ 135.64, 133.44, 127.04, 126.44, 125.58, 124.66, 123.14; GC/MS (m/z) calcd. for $C_{14}H_8S_2$ (M+) 240.01, found : 240.

(e) n-BuLi (1.4 mL, 2.25 M Solution in hexane) was added to TMEDA (0.62 mL) in dry THF (15 mL) at 0 °C under N_2 and stirring at - 78 °Cfor 30 min added dropwise compound **47** (500 mg, 2.08 mmol) in dry THF (10 mL). added dropwise DMF (0.23 mL, 3.12 mmol) at -78 °C for 2 h. The reaction mixture was quenched aq.NH_4Cl and the organic layer was separated and the aqueous layer extracted with ethyl acetate. The combined organic phases were washed with brine, dried with Na_2SO_3, and concentrated *in vacuo*. The crude residue was purified by column chromatography to give product **48** (447 g, 80 %). IR (neat, cm^{-1}) 1660.59, 1493.54, 1260.82, 1193.28, 1092.40, 1032.80, 804.78, 743.55, 725.61, 658.12, 624.87; ^1H NMR ($CDCl_3$, 300 MHz) δ 710.14(s, 1 H), 8.37(s, 1 H), 8.20(m, 2 H), 7.75(d, 1 H), 7.63(m, 3 H); ^{13}C NMR ($CDCl_3$, 500 MHz) δ 183.94, 142.19, 141.55, 136.51, 133.38, 132.88, 132.31, 128.65, 128.58, 127.01, 126.53, 126.29, 125.27, 124.77, 122.81; LC/MS (m/z) calcd. for $C_{15}H_8OS_2$ (M+) 268, found : 268.91.

(f) NBS (774 mg, 4.35 mmol) was added to a vigorously stirred solution of naphtho [1,2-b:4,3-b']dithiophene-2-carboxyaldehyde **48** (389 mg, 1.45 mmol) in 14.5 mL CH_2Cl_2 under N_2 at room temperature for 1 h. The combined organic phases were washed with brine, dried with Na_2SO_3 The solvent was evaporated under vacuum and the residue was purified by column chromatography (SiO_2, CH_2Cl_2) to furnish the product as brown solid **49** (390 mg, 77 %). IR (neat, cm^{-1}) 1666.17, 1373.29, 1233.29, 1176.45, 822.97, 746.46, 664.96, 626.15; ^1H NMR ($CDCl_3$, 300 MHz) δ 10.14(s, 1 H), 8.29(s, 1 H), 8.21(d, 1 H), 8.01(d, 1 H), 7.74(s, 1 H), 7.64(m, 2 H); ^{13}C NMR ($CDCl_3$, 500 MHz) δ 183.84, 142.47, 141.63, 137.93, 133.26, 131.86, 131.73, 128.87, 127.59, 127.29, 126.19, 125.73, 125.32, 124.45, 115.18; LC/MS (m/z) calcd. for $C_{15}H_7BrOS_2$ (M+) 345.91, found : 345.99.

(g) A mixture of naphtho [1,2-b:4,3-b']dithiophene-2-bromo-7-carboxyaldehyde **49** (150 mg, 0.43 mmol), *N*-(9,9-dimethyl-9H-fluoren-2-yl)-9,9-dimethyl-*N*-(4-(4,4,5,5-tetramethyl-1,3,2-dioxaborolan-2-yl)phenyl)-9H-fluoren-2-amine **16** (391 mg, 0.65 mmol) and $Pd(PPh_3)_2Cl_2$ (27 mg, 0.039 mmol) in 2 M aqueous Na_2CO_3 solution (1.2 mL), H_2O (0.24 mL) and 1,2-dimethoxyethane (2.15 mL) was stirred at 100 °C overnight. After cooling to room temperature, the reaction mixture was filtered through celite, and the filtrate was poured into water and extracted with CH_2Cl_2. The combined organic phases were washed with brine, dried with Na_2SO_4, and concentrated *in vacuo*. The crude residue was purified by column chromatography (ethyl acetate : hexane = 1 : 6, $R_f = 0.4$) to give adduct **50** (215 mg, 67 %). ^1H NMR ($CDCl_3$, 300 MHz) δ 10.16(s, 1H), 8.42(s, 1H), 8.22(d, J = 6.9Hz, 1H), 8.15(d, J = 6.9Hz, 1H), 7.89(s, 1H), 7.71~7.63(m, 6H), 7.42~7.40(d, J=6.3Hz, 3H), 7.36~7.28(m, 8H), 7.18~7.15(m, 3H), 1.44(s, 12H); ^{13}C NMR ($CDCl_3$, 500 MHz) δ 183.9, 155.3, 153.7, 148.5, 146.9, 145.1, 142.1, 141.6,

139.0, 135.0, 134.8, 134.8, 132.7, 132.4, 128.6, 128.5, 127.5, 127.2, 126.8, 126.7, 126.1, 125.2, 124.6, 123.7, 123.5, 122.7, 120.9, 119.7, 119.2, 117.0, 47.0, 27.2; LC/MS (m/z) calcd. for $C_{51}H_{37}NOS_2$ (M+) 743.232, found : 743.89.

(h) To a mixture of aldehyde **50** (120 mg, 0.16 mmol) and cyanoacetic acid (27 mg, 0.32 mmol) were added acetonitrile (8.0 mL) and piperidine (0.016 mL, 0.16 mmol) at room temperature. The solution was refluxed overnight. After cooling to room temperature, the organic phase was separated and the aqueous layer extracted with CH_2Cl_2. The combined organic phases were washed with brine, dried with $MgSO_4$, and concentrated *in vacuo*. The crude residue was purified by column chromatography (ethyl acetate : methanol = 1 : 1, R_f = 0.8) to give **adduct $_{t}$PS** (77 mg, 59 %) as a red solid. ^{1}H NMR (DMSO-d$_6$, 300 MHz) δ 8.70(s, 1H), 8.49(s, 1H), 8.31(s, 1H), 8.24~8.17(m, 2H), 7.83~7.75(m, 7H), 7.52(d, J = 6.9Hz, 2H), 7.35~7.25(m, 7H), 7.21(d, J = 8.7Hz, 2H), 7.12~7.09(m, 2H),1.39(s, 12H). ^{13}C NMR (DMSO-d$_6$, 500 MHz) δ 170.3, 163.0, 154.8, 153.2, 147.7, 146.2, 143.9, 139.3, 138.2, 135.2, 134.2, 133.8, 133.7, 131.7, 128.6, 127.2, 127.0, 126.8, 126.7, 125.0, 124.9, 124.3, 123.3, 122.9, 122.6, 121.2, 119.6, 118.8, 118.3, 116.9, 46.4, 26.6; LC/MS (m/z) calcd. for $C_{54}H_{38}N_2O_2S_2$ (M+) 810.24, found : 810.64.

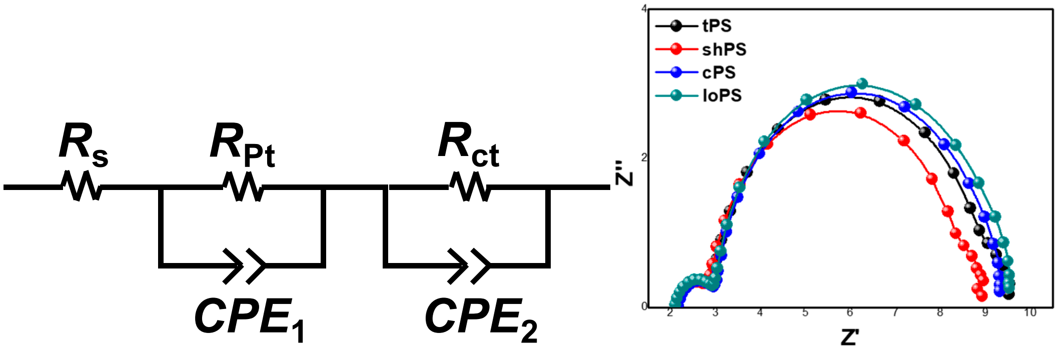

Figure S7. Equivalent circuit used in electrochemical impedance analysis.

Table S1. Photovoltaic performance of DSSCs with PS series metal free organic dyes.

Dye	J_{sc} (mA/cm^2)	V_{oc} (mV)	*FF*	η(%)
$_{sh}$PS	7.86	846.6	0.73	4.84
	7.92	835.2	0.73	4.81
	7.47	846.2	0.73	4.60

	7.65	835.9	0.72	4.59
Average	7.73±0.23	840.1±5.7	0.73±0.01	4.71±0.13
$_{lo}$PS	6.38	736.4	0.67	3.16
	6.69	736.8	0.67	3.32
	6.47	740.8	0.67	3.22
	6.76	743.5	0.66	3.33
Average	6.58±0.19	739.4±3.55	0.67±0.01	3.26±0.09
$_{c}$PS	7.77	743.1	0.72	4.14
	7.69	743.8	0.72	4.10
	7.01	735.1	0.72	3.69
	7.78	745.9	0.71	4.10
Average	7.56±0.39	742.0±5.4	0.72±0.04	4.00±0.23
$_{t}$PS	5.27	832.8	0.66	2.90
	5.27	846.1	0.65	2.90
	5.28	831.9	0.66	2.90
	5.17	845.6	0.65	2.85
Average	5.25±0.03	839.1±6.5	0.66±0.01	2.89±0.01
